# NLPR3 Inflammasomes and Their Significance for Atherosclerosis

**DOI:** 10.3390/biomedicines8070205

**Published:** 2020-07-10

**Authors:** Anastasia V. Poznyak, Alexandra A. Melnichenko, Reinhard Wetzker, Elena V. Gerasimova, Alexander N. Orekhov

**Affiliations:** 1 Institute for Atherosclerosis Research, Skolkovo Innovative Center, 121609 Moscow, Russia; 2 Laboratory of Angiopathology, Institute of General Pathology and Pathophysiology, 125315 Moscow, Russia; sasha.melnichenko@gmail.com; 3 Department of Anesthesiology and Intensive Care Medicine, Jena University Hospital, 07747 Jena, Germany; Reinhard.Wetzker@uni-jena.de; 4 V.A. Nasonova Institute of Rheumatology, 34A Kashirskoye Shosse, 115522 Moscow, Russian; gerasimovaev@list.ru; 5 Institute of Human Morphology, 3 Tsyurupa Street, 117418 Moscow, Russia

**Keywords:** inflammasome, atherosclerosis, CVD, NLRP3 inflammasome

## Abstract

Atherosclerosis is a serious disorder, with numerous potential complications such as cardiovascular disease, ischemic stroke, and myocardial infarction. The origin of atherosclerosis is related to chronic inflammation, lipid metabolism alterations, and oxidative stress. Inflammasomes are the cytoplasmic multiprotein complex triggering the activation of inflammatory response. NLRP3 inflammasomes have a specific activation pathway that involves numerous stimuli, including a wide range of PAMPs and DAMPs. Recent studies of atherosclerotic pathology are focused on the mitochondria that appear to be a promising target for therapeutic approach development. Mitochondria are the main source of reactive oxygen species (ROS) associated with oxidative stress. It was previously shown that NLRP3 inflammasome activation results in mitochondrial damage, but the exact mechanisms of this need to be specified. In this review, we focused on the features of NLRP3 inflammasomes and their significance for atherosclerosis, especially concerning mitochondria.

## 1. NLRP3 Inflammasome

The inflammasome is a cytoplasmic multiprotein complex that promotes the activation of inflammatory response. Activated inflammasomes contribute to the release of mature cytokines, and thus to the innate immunity response formation. They consist of a sensory protein, and an adapter protein (an apoptosis-associated speck-like protein) [1].

Different sensory proteins within the inflammasome complex are triggered by various stimuli, such as microbial products and host-danger signals, which leads to the gathering of the inflammasome summation. This process is realized through activation and enrollment of the caspase domain (CARD) and homotypic interaction of the pyrin domain (PYD) [2,3].

NLRP3 inflammasomes differ from other inflammasome types by the activation mechanism. They are activated by exogenous or endogenous damage-associated molecular patterns (DAMPs), and are involved in the sterile inflammation [4]. NLRP3 inflammasome consists of NLRP3, ASC, (apoptosis-associated speck-like protein) and caspase-1 proteins. ASC contains an N-terminal PYD and a C-terminal caspase recruitment domain (CARD). NLRP3 contains three domains: C-terminal leucine-rich repeats (LRR), a central nucleotide domain called the NACHT domain, and an N-terminal effector domain (pyrin domain [PYD]). Caspase-1 has conservative domains for homophilic interaction, and it also contains CARD and catalytic domains [5].

**Scheme 1:**
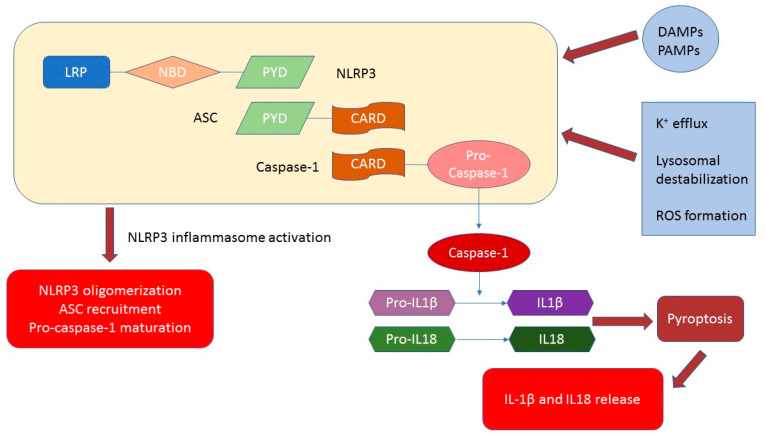
NLRP3 inflammasome structure and activation. The main triggers of activation are pathogen-associated molecular patterns (PAMPs) and damage-associated molecular patterns (DAMPs). Additionally, potassium efflux, lysosomal destabilization, reactive oxygen species (ROS) formation and other extra- and intracellular stimuli contribute to the NLRP3 inflammasome activation.

NLRP3 is assembled by the NACHT domain during stimulation with DAMP, so that, through their interaction between PYDs, it promotes ASC oligomerization. Due to CARD’s lack, Caspase-1 cannot be recruited. However, this process can offset the presence of ASC in NLRP3. Then, through the homophilic interaction of CARD, the oligomerized ASC induces self-activation of caspase-1 and also interacts with it [6]. Scheme of NLRP3 activation is shown in Scheme 1.

As a result of this process, proinflammatory effects occur. Moreover, caspase-1 produces pyroptosis by cleaving gasodermin D (GSDMD), and it processes pro-IL-18 into its more mature biologically active form. This process is accompanied by increased permeability of the plasma membrane [7,8]. Pyroptosis-mediated IL-1 membrane permeabilization is crucial for the release of IL-1, and does not have a signal sequence for exocytosis. Nevertheless, the release mechanism of IL-1 has not been fully revealed. Extracellular adenosine triphosphate (ATP), monosodium triurate (MSU), silica, and other exogenous and endogenous signals of danger activate the inflammatory processes of NLRP3. However, the exact recognition mechanism of NLRP3 DAMP is still unclear [9]. 

Potassium efflux, lysosomal destabilization, and the formation of mitochondrial reactive oxygen species (ROS) constitute the general ascending pathways that are important for the activation of the NLRP3 inflammasomes [10]. Thus, lysosomal destabilization and the following extrication of cathepsin are in charge of the activation of inflammation via NLRP3 particles. 

The process of synthesis and the release of IL-1 can be described in two-steps [11]. Firstly, it is a transcriptional synthesis of pro-IL-1. Then, inflammasomes turn the pro-IL-1 from proteolytic into a mature form. The transcriptional control of IL-1 mRNA is regulated by PRRs or cytokine receptors, and is known as priming. Among these receptors are IL-1 receptor (signal 1) and TLRs, and NF-kB-mediated mRNA stimulation of IL-1 and NLRP3. Triggering of these receptors was demonstrated to prime the NLRP3 by posttranscriptional control, as ubiquitination and deubiquitination [12]. Hereupon, the assembled pro-IL-1 in the cytosol is quickly processed by activated caspase-1. Caspase-1 is activated by NLRP3 inflammasomes also known as signal 2. Accordingly, such a two-step scheme is essential for tight moderation of the forceful inflammatory cytokine IL-1 to support inflammatory homeostasis [13].

## 2. Significance for Atherosclerosis

Atherosclerosis is a disease related to inflammation and lipid metabolism alterations. The formation of inflammatory plaques in the walls of blood vessels is a specific sign of atherosclerosis. Such plaques are described by the infiltration of inflammatory cells and the accumulation of lipids [14].

Hypercholesterolemia is the key risk factor for atherosclerosis. In the process of forming plaques, crystalline cholesterol and oxLDL are engaged extensively. 

The significant feature of the inflammatory reply is that LDL undergo numerous modifications that give the atherogenic properties to the particles. Among such modifications, there are desialylation, oxidation, and changes in size and density of LDL [15]. 

Among risk factors of atherosclerosis, there are hypercholesterolemia, hypertension, diabetes, and smoking. In 2013, scientists showed overexpression of NLRP3 in the aorta of smoker patients with hypertension, diabetes, and hypercholesterolemia [16]. For those patients, the expression of NLRP3 in the aorta was connected with LDL-cholesterol, total cholesterol, and lipoprotein (a), and inversely associated with HDL-cholesterol levels. 

**Scheme 2:**
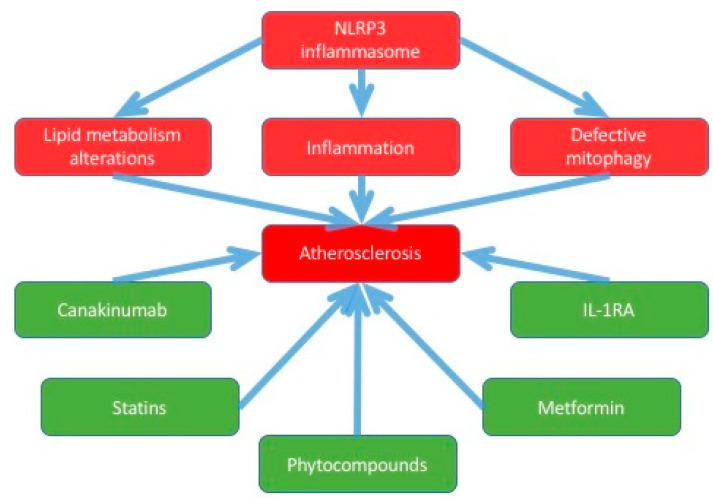
This scheme shows the associations between atherosclerosis and NLRP3 inflammasomes, and the potential compounds for the therapy.

Every mechanism of inflammasome activation is present in atherosclerosis: mitochondrial dysfunction, ER stress [17], lysosome rupture, and oxidative stress. Moreover, all crucial for atherogenesis processes were shown to be associated with NLRP3 inflammasome (see Scheme 2).

The process of atherosclerosis is characterized by, firstly, released ROS by lipid oxidation and oxidative stress. Then, ROS provoke transformation LDL and HDL to ox-LDL and ox-HDL. This process results in the secretion of ROS. Its downstream cytokines release and activate the NLRP3 inflammasome [18].

A few studies have demonstrated the important function of the NLRP3 inflammasome for the pathogenesis of atherosclerosis. NLRP3 corresponds to the probability of coronary atherosclerosis in patients with acute coronary syndrome [19]. It has also been noticed that NLRP3 inflammasomes are located in the cytoplasm of macrophages and foam cells [8].

It was shown that ox-HDL, ox-LDL, and HDL regulate activation of the NLRP3 inflammasome and downstream cytokines, and thus take part in the pathogenesis of atherosclerosis [20]. Nevertheless, further studies are required for a full understanding of such a mechanism. There are several hypotheses considering the process of NLRP3 inflammasome activation [21]. 

The first hypothesis [22] demonstrated that activation of the NLRP3 inflammasome by cholesterol crystals induced dose-dependent secretion of mature IL-1β. This is released through the lysosomal protease cathepsin B outflow to the cytoplasm that is triggered by the cholesterol crystals. Inhibition of the lysosomal protease cathepsin B, in turn, decreases the IL-1β release [23].

The same process is observed for an inhibitor of cathepsin B: CA-074 remarkably reduces IL-1β secretion [24]. To activate the NLRP3 inflammasome, the lysosome damage and the leakage of lysosomal cysteine protease cathepsin B into the cytoplasm were inferred [25,26]. 

The second version demonstrated that caspase-1 recruitment and the NLRP3 inflammasome assembly reply may be suppressed at high extracellular potassium concentrations in Thp-1 macrophages. It may propose that potassium efflux may also activate NLRP3 [27].

The third mechanism suggested to mediate NLRP3 inflammasome activation involves ROS production [28]. The authors propose that ROS are abundantly produced by the stimulation of many NLRP3 activators. The inflammatory responses may be accompanied by the suppression of ROS [20].

Finally, the study demonstrated that NLRP3 inflammasome control signaling regulates ox-LDL-induced inflammation. Then, ox-HDL provokes the inflammation by activating the NLRP3 inflammasome, which has a significant function in the progression of atherosclerosis. 

Notably, the HDL has a protective effect: it inhibits the expression of NLRP3 and its downstream cytokines. Last but not least, interference with the key components of the NLRP3 inflammasome could become another therapeutic approach to atherosclerosis [29]. 

At the same time, it has been proposed that not NLRP3 but NLRP1 has a significant function in switching the endothelial cells to the proinflammatory condition [30]. 

### 2.1. Relation with Mitochondrial Damage

Mitochondrial events and NLRP3 inflammasome activation seem to be tightly bounded. The NLRP3 inflammasome may result in mitochondrial damage through mtROS [31]. The growing body of evidence supports the following concept: the refining of mtROS effectively inhibits the activation of the NLRP3 inflammasome [32]. According to that surveillance, weakened mitophagy was demonstrated to strengthen the activation of the NLRP3 inflammasome. This process is caused by an insufficiency to shake off damaged mitochondria. 

Mitochondrial DNA (mtDNA), which is released into cytoplasm from the damaged mitochondria, has also been suggested to operate as a mitochondrial signal of danger. Such signal activates NLRP3 [33]. Those discoveries share the idea that the dysfunction of mitochondria might contribute to the assembly of the NLRP3 inflammasome. Although recent data proposed that activation of the NLRP3 inflammasome is connected with mitochondrial dysfunction [34], the function of NLRP3 activators in causing mitochondrial damage remains unknown. 

The potential trigger might be a growth in an intracellular Ca2+ degree. NLRP3 stimulators (ATP and others) may result in Ca2+ inflow and contribute to mitochondrial damage, leading to the depletion of mitochondrial membrane potential and the production of mtROS [35]. Moreover, Yaron et al. demonstrated how K+ expiration controls the influx of Ca2+. Then, it induces the overburden of mitochondrial Ca2+ and culminates in mitochondrial dysfunction [36]. On the other hand, different research suggested that K+ efflux produces NLRP3 activation in a Ca2+ influx-independent manner [37]. However, further studies are needed to fully understand the significance of Ca2+ flux in NLRP3 inflammasome activation and mitochondrial dysfunction and its mechanism. 

Lately, the other kind of mitochondrial mutation connected to NLRP3 inflammasome activation was described. Scientists have shown that NLRP3 is activated by N-acetylglucosamine obtained from peptidoglycan of G+ bacteria, separating hexokinase from mitochondria outer membrane [38]. Thus, a connection between inflammatory and metabolic processes is suggested.

There are also contradictory outlines about the causality between NLRP3 activation and mitochondrial damage. Yu et al. suggested that inflammasome activation results in mitochondrial damage [39], proposing that mitochondrial damage may not be the cause but the consequence of inflammasome activation. Besides, the exact target of mtROS in NLRP3 activation remains undiscovered. Some scientists propose that ROS scavengers (as N-acetyl-lysine (NAC)) do not influence the direct activation of NLRP3 by classical NLRP3 agonists, although they stop the transcription of NLRP3 and pro-IL-1β [40]. 

### 2.2. NLRP3 Inflammasome and Lipids

LDL oxidation is one of the numerous atherogenic modifications that happen within the blood flow. Oxidized LDL (oxLDL) is impervious to the lysosomal enzymes of macrophages, which result in the oxLDL storage in macrophages and successive formation of foam cells [41]. OxLDL ties CD36 and is phagocytosed by macrophages. The precise mechanism of LDL oxidation is controversial, and several oxidative sources such as ROS, metal ions, lipoxygenase, and myeloperoxidase have been suggested [42,43]. On the other hand, a considerable number of studies have proposed the impact of oxLDL on the development of atherosclerotic plaques. Since cholesterol crystals and oxLDLs cause inflammasome activation, inflammasome connects the inflammatory condition and lipid metabolism. 

Consequently, oxLDL may supply signals needed for the activation of inflammasome in atherosclerosis. Then, the macrophage priming via TLRs that activate NLRP3 and formation of cholesterol crystals takes place. In the low-density lipoprotein receptor (LDLR)-deficient mouse model of atherosclerosis, NLRP3-deficient mice were kept safe from disease, obviously involving NLRP3 [44]. Since atherosclerotic plaques have a low pH, this could be a significant element for the activation of the inflammasome. Furthermore, Raiamaki et al. revealed that extracellular acidosis triggers NLRP3 [45]. Besides, another study discovered that NLRP3 in endothelial cells in atheroprone areas of the aorta might be activated by oscillatory flow [46]. Presumably, it is controlled by the activation of ROS and SREBP2, which successively trigger and then activate NLRP3. 

It was also demonstrated that cholesterol activates the NLRP3 inflammasome. What is more, the layer of cholesterol crystals can be found in advanced plaques. When cholesterol crystals are taken up by macrophages, they become potent NLRP3 agonists. This was shown to occur in a complement-dependent way [47]. Moreover, oxLDL can activate NLRP3 actually sans preformed crystals [48]. The procedure includes CD36-mediated absorption of oxLDL by macrophages, which result in the intracellular nucleation of cholesterol crystals in lysosomes [49]. Such a process activates the inflammasome identically, as if the crystals were taken up by phagocytosis. 

### 2.3. Proinflammatory Cytokines

Patients undergoing carotid endarterectomy showed enhanced levels of NLRP3; ASC; and caspase-1, IL-1β, and IL-18, in the unstable carotid atherosclerotic plaques [20].

Apolipoprotein E-deficient (apo E−/−) mice fed with a high-fat diet demonstrated the deceleration of NLRP3 signaling by lentivirus-mediated RNA interference, which suppresses proinflammatory cytokines. Such RNA interference diminished the amount of lipids and macrophages and raised SMCs and collagen contents of the plaque. This process results in plaque stabilization [16].

Moreover, there is an inflammasome activator, located in the necrotic parts of atherosclerotic plaques–pyroptosis, extracellular Ca2+. IL-1β and IL-18 are the products of inflammasome activation. They have a significant function in the atherogenic process.

IL-1β production in endothelial cells and macrophages occurs in coronary arteries with atherosclerotic lesions. Furthermore, for apo E−/− and IL-1β−/− mice compared with the control group of apo E−/− mice, nearly 30% decline in the atherosclerotic plaques size has been noticed. For apo E−/− mice, monoclonal antibodies against IL-1β suppress plaque formation [50]. Reduced levels of IL-1β are followed by the reduced degree of inflammatory molecules as VCAM-1, IL-6, IL-8, MCP-1, TNF-α, MMP-3, and MMP-9. IL-18 is a proatherogenic and proinflammatory cytokine. Upregulation of IL-18 and its receptor takes place in endothelial cells, macrophages, and SMCs. Moreover, the expression of IL-18 in higher in nonstable plaques. In contrast to IL-18 competent littermates, investigations in apo E−/− and IL-18−/− mice demonstrated a reduction of IL-18 in plaque by 35% [50].

Pyroptosis occurs during activation of caspase-1 by oxLDL in macrophages [51]. Pyroptosis is probably engaged in the progress of atherosclerotic plaque formation, since it intensifies the inflammation [52]. 

Moreover, ASK- 1 and JNK-1 take part in inflammasome activation and weaken plaque formation by producing apoptosis in macrophages. These are upstream molecules in caspase-8 activation, which cause apoptosis [53]. Activation of caspase-8 in macrophages may induce apoptosis by the NLRP3 inflammasome. It is yet an unanswered question if NLRP3 inflammasome mediates the JNK and ASK-induced apoptosis, or whether at the lower intensity of stimuli the NLRP3 inflammasome apoptotic role saves against atherosclerosis development. 

## 3. Targeting NLRP3 Inflammasome for the Treatment of Atherosclerosis

NLRP3 inflammasomes play an important role in the pathogenesis of atherosclerosis and thus appear to be the promising target for the therapeutic approach. Many already established compounds were tested for the ability to modify NLRP3-related pathways. Most promising of them are shown on the Scheme 1 and also described below. 

### 3.1. Statins

Statins are known for their ability to inhibit cholesterol synthesis, which is beneficial for atherosclerosis treatment. Additionally, their anti-inflammatory properties were described [54]. 3-hydroxy- 3-methyglutaryl coenzyme A inhibitor, known as atorvastatin, was shown to lower the production of NLRP3, IL1, and caspase-1 in PMA-induced THP-1 cells. Atorvastatin exhibited anti-inflammatory properties and was demonstrated to inhibit the PMA-induced THP-1 monocyte via the TLR4/MyD88/NF-KB pathway [55].

Simvastatin was also shown to lower the risk for cardiovascular patients. This compound also contributed to the lowering of IL1 [56].

### 3.2. Metformin

Metformin was showed to reduce both the expression of NLRP3 and activation of NLRP3 inflammasome. The mechanism of this inhibition is implemented through the enhanced expression of adenosine monophosphate-activated protein kinase (AMPK) and protein phosphatase 2A (PP2A) [57]. Moreover, metformin is known to reduce the glucose level, which is also potentially contributes to a decreased rate of NLRP3 inflammasome activation. In vitro investigations revealed the decreased activation rate of NLRP3 inflammasome in the ApoE−/− mice model and inhibited atherosclerosis in diabetes [58].

Colchicine and dapagliflozin were also shown to have a beneficial effect on atherosclerosis, which is potentially implemented through the inhibition of NLRP3 inflammasome activation [54,59].

Melatonin is able to inhibit expression of IL-1β, NLRP3, cleaved caspase-1, and other proteins associated with the aortic endothelium. It was also shown that melatonin acts via stimulation of mitophagy and inhibition of the NLRP3 inflammasome activation through the Sirt3/ FOXO3a/Parkin signaling pathway [60].

### 3.3. Phytocompounds

Not only synthetic compounds but even natural ones were shown to be effective in every way against atherosclerosis.

For example, artemisinin showed the ability to suppress the inflammatory response via the AMPK/ NF-κB-NLRP3 pathway in macrophages and thus exhibit vascular protective properties [61].

Curcumin was demonstrated to inhibit TLR4/MyD88, NF- κB, and P2X7R in phorbol 12-myristate 13- acetate-induced macrophages, which resulted in decreased activation of NLRP3 inflammasomes. The expression of NLRP3, caspase-1, and IL-1β was decreased in the presence of curcumin [62].

Rosmarinic acid is another example of a natural compound with antiatherogenic properties. Activation of pyrin domains of NLRP3 inflammasome appeared to be lower in response to rosmarinic acid, and the production of ROS after nicotine was involved in vascular smooth muscle cells [63].

### 3.4. Clinical Trials Targeting NLRP3 Pathway in Atherosclerotic Patients

For now, there are no available data on the use of inflammasome targeting in the treatment of atherosclerosis or even any other cardiovascular disorders. However, several clinical trials have been conducted. For example, Abbate et al. conducted randomized double-blind pilot trials for patients with acute myocardial infarction (MI) [64]. This research demonstrated that the IL-1 receptor antagonist (IL1RA) positively affected left ventricular remodeling and was safe. On the other hand, for IL-1RA in patients with non-ST elevation acute coronary syndromes, short-dated treatment (two weeks) was connected with a considerable surplus of significant adverse cardiovascular events after 1-year of follow up [65] and did not show a stable lowering of inflammatory markers levels.

Canakinumab Anti-inflammatory Thrombosis Outcomes Study (CANTOS trial) assessed the influence of IL1β blockade on the occurrence/recurrence of cardiovascular events among 17,200 stable patients at high vascular risk, diagnosed with CAD (coronary artery disease) [66]. Canakinumab is a human monoclonal antibody that selectively neutralizes IL-1β.

Results that showed a comparison of active doses of canakinumab and placebo were assessed.

Initial information already maintained the usage of the anti-IL-1β antibody as a possible therapeutic approach, since it considerably lowers the inflammation rate [67]. However, it did not influence vascular function in those patients or vascular structures [68]. As a result of the trials, the reduction of cardiovascular death, the risk of major adverse cardiovascular events, non-fatal stroke, and non-fatal myocardial infarction were reported [66].

Canakinumab therapy was connected with a downfall of fatal infection in comparison to placebo. This test extends public comprehension of the mechanism of the balance of innate immunity that contributes to cardiovascular health, which provides safety and efficacy data on long-term suppression of IL-1β dependent immunity [66].

## 4. Conclusions

NLRP3 inflammasome activation was proven to participate in numerous processes related to atherosclerosis and cardiovascular disease. Thus, oxidative stress, inflammation, lipid metabolism, and mitophagy, which are crucial for atherogenic processes, involve pathways related to NLRP3 in various ways. This broad impact makes inflammasome the promising object of further investigations of atherosclerosis and a promising potential target for antiatherogenic treatment.

However, further studies are needed to understand all molecular mechanisms in detail.

To date, various compounds were tested for their ability to inhibit the development of atherosclerosis. However, none showed absolute efficacy.

Such well-known compounds as statins, colchicine, and metformin were shown to modify the NLRP3-related pathways. Anti-inflammatory properties of these drugs are implemented, at least in part, by the reduction of NLRP3 and IL1 production and also by lowering of the NLRP3 inflammasome activation rate. Moreover, CANTOS trials resulted in a decreased number of cardiovascular deaths, the risk of major adverse cardiovascular events, non-fatal stroke, and non-fatal myocardial infarction in response to the use of canakinumab, which acts as the selective inhibitor of IL1. These data provide a body of evidence for the outstanding potential of targeting NLRP3 inflammasome-related pathways.
